# Data mining methodology for obtaining epidemiological data in the context of road transport systems

**DOI:** 10.1007/s12652-022-04427-2

**Published:** 2022-10-01

**Authors:** Teresa Cristóbal, Alexis Quesada-Arencibia, Gabriele Salvatore de Blasio, Gabino Padrón, Francisco Alayón, Carmelo R. García

**Affiliations:** Institute for Cybernetics, University of Las Palmas de Gran Canaria, Campus de Tafira, 35017 Las Palmas de Gran Canaria, Spain

**Keywords:** Intelligent transport systems, Data mining, Network epidemiology, Contact patterns, COVID-19

## Abstract

Millions of people use public transport systems daily, hence their interest for the epidemiology of respiratory infectious diseases, both from a scientific and a health control point of view. This article presents a methodology for obtaining epidemiological information on these types of diseases in the context of a public road transport system. This epidemiological information is based on an estimation of interactions with risk of infection between users of the public transport system. The methodology is novel in its aim since, to the best of our knowledge, there is no previous study in the context of epidemiology and public transport systems that addresses this challenge. The information is obtained by mining the data generated from trips made by transport users who use contactless cards as a means of payment. Data mining therefore underpins the methodology. One achievement of the methodology is that it is a comprehensive approach, since, starting from a formalisation of the problem based on epidemiological concepts and the transport activity itself, all the necessary steps to obtain the required epidemiological knowledge are described and implemented. This includes the estimation of data that are generally unknown in the context of public transport systems, but that are required to generate the desired results. The outcome is useful epidemiological data based on a complete and reliable description of all estimated potentially infectious interactions between users of the transport system. The methodology can be implemented using a variety of initial specifications: epidemiological, temporal, geographic, inter alia. Another feature of the methodology is that with the information it provides, epidemiological studies can be carried out involving a large number of people, producing large samples of interactions obtained over long periods of time, thereby making it possible to carry out comparative studies. Moreover, a real use case is described, in which the methodology is applied to a road transport system that annually moves around 20 million passengers, in a period that predates the COVID-19 pandemic. The results have made it possible to identify the group of users most exposed to infection, although they are not the largest group. Finally, it is estimated that the application of a seat allocation strategy that minimises the risk of infection reduces the risk by 50%.

## Introduction

One of the main routes for the transmission of infectious diseases is human-to-human contact. Of these, respiratory diseases affect thousands of people every year. COVID-19 is an example of such a disease, caused by a pathogen with a high rate of propagation, which is why the World Health Organization (WHO) declared a global pandemic in March 2020. The WHO^1^ states that any situation involving close proximity between people for a long period of time increases the risk of transmission. In the context of a health crisis caused by this type of disease, it is important to understand the patterns of social contact or proximity between people, as this information is used to implement effective epidemiological control measures (Hoang 2019).

The mission of transport systems is to facilitate the mobility of people; millions of people around the world move through their infrastructures every day.^2^ For this reason, such systems have attracted scientific interest in the epidemiology of airborne diseases (Troko 2011). This article describes a methodology for obtaining epidemiologically relevant data, as well as the patterns that these data follow, to help both transport operators and health authorities to develop effective health control measures based on the usage data of an intercity road transport system. The ultimate goal of the methodology is to provide epidemiological data that will help health authorities to implement effective epidemiological control measures such as the identification of target population groups in vaccination campaigns based on the mobility and contact patterns of these groups, or non-pharmacological measures such as travel restrictions. In the context of road transport systems, and in line with the points made by (Tirachini 2020) on the challenges that transport systems need to address in order to make them safer from an epidemiological point of view, these data would also be useful for transport operators and transport agencies when making decisions to reduce the risk of infection among passengers. To achieve this goal, a data mining methodology was developed to analyze the millions of data records that are generated by the trips made by its users.

Since the beginning of this century and especially with the emergence of new infectious respiratory diseases, such as SARS and COVID-19, the use of data analytics in systems based on data mining and big data has been proposed as a useful resource to help in predicting, tracking, monitoring and decision-making in the epidemiological control of this type of disease (Corsi 2021). The methodological proposal presented herein belongs to this general framework. In a more specific context of transport systems and epidemiology, there are two methodological precursors to this proposal. The first is the work of Eubank (2004) which predicted the propagation of an infectious disease and analysed the impact that different epidemiological control strategies would have on a population, using mobility data to generate a graph of the co-presence of people in the population under analysis and to run simulations. The second precursor is the work of Goscé (2018) which, by analysing the data provided by the contactless card-based payment system used by users of a metropolitan transport network, analysed their mobility with the aim of identifying the relationship between crowded spaces in the transport network and the spread of the disease. The proposed methodology combines these two approaches. In our particular study, we make three main contributions:

- The first is that, to the best of our knowledge, there are no previous published works on obtaining epidemiologically relevant data and their patterns in the context of road transport systems, or if there are, they are very limited.
- Second, the proposed methodology. It is a novel proposal intended to systematically obtain data that are relevant for epidemiological monitoring. Starting with a formalization of the problem, based on concepts related to epidemiology and transport activity, the methodology obtains these data by estimating the number of close contacts between passengers in the transport system.
- And third, the findings provide information that can be used for epidemiological control in pandemic situations. This is particularly relevant because urban dwellers spend 7% of their time travelling on transport systems (Jenkins 1992).

In addition to this introduction, this paper contains five more sections. The following section presents work related to the topics addressed in this paper. The methodology is then described in the third section. The fourth section presents the results and discussion obtained by applying the proposed methodology to a real case of a road transport system. The limitations of the study is presented in section fifth and finally, the sixth section presents the conclusions.

## Related works

Network theory is used in many fields of knowledge, including epidemiology. This is because interactions between people can be modelled by means of a network called a contact network, where contact – in this field – is understood to mean interaction between two or more people that may lead to the transmission of a disease. There is extensive literature on the use of network theory in epidemiology that can be organized into two types: studies on the use of inferred contact networks, based on social contact surveys or human behavior simulations, and those on the use of contact networks generated from proximity sensor data (Danon 2010). The methodologies followed by all these studies have the same objective, which is to obtain data useful for modelling the dynamics of infectious disease, using mathematic models such as those based on the next-generation matrix (Diekmann 1990) or the compartmental SIR (susceptible–infected–recovered) model (Kendall 1956), or to evaluate the impact of epidemiological control measures. These data are: frequency of contacts, duration, location of contacts, contact network, and contact matrices to represent the contact network between different clusters of participants. In this section, three types of studies are reviewed: on the use of inferred contact networks, on the use of contact networks generated from proximity sensor data and finally, on the role of transport systems in the transmission of this type of disease. Because they do not coincide with the aims of the research presented in this article, we have not considered studies on the tracing of contacts for epidemiological control in health crisis situations.

### Studies based on inferred contact networks

A methodology for obtaining patterns of social contacts between individuals is to use census data, assuming that they reflect the distribution of groups in the population and household size (Ferguson 2005; Longini 2005). A more realistic methodology is to derive these patterns from data obtained from surveys of the populations under study. Wallinga (2006) presented a study on how to obtain transmission parameters by age group from a social contact survey on the conversational partners of the participants. The survey was conducted by face-to-face interviews in Utrecht, the Netherlands, in 1986, where 3084 were invited, 2106 completed a questionnaire and 1813 met the criteria for further analysis. A survey-based study involving 7297 participants from 8 European countries is presented in Moosong (2008). The results of this study show that the patterns of social contacts in the different countries were very similar to each other, and that social contact tended to take place between people of the same age, especially in the population groups of schoolchildren and young adults. This study was conducted in the framework of the European POLYMOD project. With a view to building on the contact patterns obtained in the POLYMOD project, the BBC Pandemic project (Klepac 2018) was developed in the United Kingdom and reported social contact information from 40,177 participants who completed the study, out of the 86,000 participants initially recruited. A considerable number of studies were conducted along the same lines in specific geographical settings. A review of these studies is presented in Hoang (2019). As a result of this comprehensive review, the authors proposed various recommendations that should be taken into account for future survey-based work.

Simulation of human behavior is another alternative technique that has been used in studies on the use of networks in epidemiology. In order to better predict outbreaks of SARS, using different mathematical models, in Meyers (2005) the contact network of an urban population was obtained through a stochastic simulation of the behavior of the people in the population, where contacts occur randomly in homes, schools, workplaces, hospitals and other public places. Stochastic simulation of the behavior of individuals belonging to large populations was also used in Chao (2010). The researchers found that the dynamics of influenza epidemics modelled using the contact network generated from the simulation was consistent with epidemiological data from the 1957–1958 and 2009 influenza pandemics.

### Studies based on contact data collected via proximity sensors

The following is a review of literature on contact networks generated in different contexts of social relationships, using different types of sensors. In Isella (2011a), contact data from two social events were analyzed: a scientific conference and a museum exhibition. The contact data were obtained from proximity records provided by RFID tags on badges worn by participants at these events. At the scientific conference, 100 people participated, registering around 10,000 contacts, and at the exhibition, 14,000 people participated, registering more than 230,000 contact records. A framework for recording proximity contacts in different social contexts is presented in Cattuto (2010). The technology used in this proposal is RFID and its objective is to have a scalable, high-resolution environment that generates the contact network in different types of social contexts. This paper presents the results obtained by using the proposed framework at three different social events: an exhibition in which 25 people participated and 8700 contacts were recorded, and two scientific conferences. In the first, 575 people participated and 17,000 contacts were recorded, and in the second, 405 people participated and 60,000 contacts were recorded. Salathé (2010) describes how a network of proximity interactions (up to 3 m) was obtained in a high school. To obtain the close proximity records, the researchers used a mobile sensor network based on TelosB motes. The number of contacts recorded was 2,148,991 and 788 people participated in the study, covering 94% of the entire school community, and the study was conducted in the month of January. Isella (2011b) describes a study conducted to obtain a network of close contacts (up to 1.5 m) between patients, healthcare staff and caregivers in a pediatric ward. The technology used in this study was RFID, with each participant provided with an RFID tag. The number of contacts recorded was 16,000, obtained during 7 days in November 2009, the peak period of the 2009 A/H1N1 influenza pandemic. In Stehlé (2011), a study was conducted to obtain a network of close contacts between students and teachers in a primary school. The study involved 232 children and 10 teachers and recorded their close contacts (up to 1.5 m) on 1 and 2 October 2009. The technology used was RFID and a total of 77,602 contact events were recorded. Stopczynski (2015) analyzes how proximity between people affects the spread of an infectious disease, using an SIR (susceptible-infected-recovered) model. The authors used two networks: one of short-distance interactions (≤ 1 m) and one of long-distance interactions (≤ 10 m). Around 500 students from the Technical University of Denmark participated in this study, which used Bluetooth technology to record the proximity between them. Génois (2019) analyzed the properties of contact networks at different spatial resolutions, studying the differences between real face-to-face contact networks and face-to-face contact networks estimated from co-presence data. Finally, in the context of the COVID-19 pandemic, Kumar (2021) and Barnawi (2021) proposed systems based on unmanned aerial vehicles (UAVs) equipped with image sensors for estimating the distance between people in densely crowded places.

### Epidemiological studies in the context of transport systems

This section reviews epidemiological studies that have used data from different transport systems as their main source of data. The aim of these studies is to analyze the role that transport systems have played in the spread of an infectious respiratory disease. In Eubank (2004), an agent-based simulation is used to model the movements of 1.5 million people in Portland, Oregon, USA, between 180,000 different locations. The goal of the simulation was to detect the presence of two people in the same place at the same instant in time, to generate a static network of contacts, and to predict the number of contacts that occur at each location. Colizza (2006) studies the role of passenger flows in air transport networks in predicting the spread of a pandemic. Merler (2010) analyzes the evolution of an influenza epidemic on a European scale, using data from the European railway system as a source for modelling long-distance mobility, exploring the role of population heterogeneity and human mobility in the spread of the pandemic. Balcan (2009) analyzes the role of different transport networks in the spread of an epidemic when their mobility flows are integrated and they operate at different scales. This paper analyzes the interplay between small-scale mobility flows in ground transport and large-scale mobility patterns in air transport in shaping the spatiotemporal patterns of a global influenza epidemic. Cooley (2011) presents a computer simulation for analyzing the evolution of an influenza epidemic in New York, based on an SEIR (susceptible–exposed–infectious–recovered) influenza disease transmission dynamics model, using epidemiological parameters obtained from the 1957 to 1958 influenza pandemic, and using the subway ridership data of 7,847,465 virtual people. Information on each person included sex, age, employment status, occupation, income, location of residence and members of the household. The results showed that if a flu epidemic with the same characteristics as the 1957–1958 epidemic were to occur, 4% of the infections would occur on the subway, compared to 30% at home, 37% at work (or school) and 33% in community activities outside the home and work (or school). Goscé (2018) presents a study on the relationship between the crowded environments of public transport systems and the spread of airborne diseases. The study is based on mobility data from the London Underground, specifically trip data generated by its passengers using an automatic payment system based on a contactless card. These data were used to study crowd dynamics at different stations. The model used to estimate the spread of the disease takes into account the route followed by each passenger, with the probability of being infected varying depending on the estimated level of crowding in the different places through which the passenger passes. The results of this study show that, in boroughs with a higher incidence of this type of illness, residents spend more time in the Underground. The authors therefore suggest that research is needed to quantify the role of public transport use in the transmission of this type of illness.

In the specific context of the COVID-19 pandemic, Luo (2020) describes a contact-tracing study on an outbreak in Hunan Province, China, involving 10 passengers on two public transport buses. Based on the limitations and results of this study, the authors suggest that COVID-19 infections are caused by aerosol transmission due to poor ventilation in the vehicles. Considering the results of this publication, to reduce the risk of infection, a series of measures should be taken, such as: disinfecting vehicles regularly, ventilating vehicles adequately, opening windows when possible, and transport users wearing face masks and maintaining hand hygiene. Shen (2020) presents a case of community transmission among attendees of a religious event in Zhejiang province (China). In this case, 28 of the 68 people affected took the same bus to the event. The authors suggest that the cause of this outbreak was airborne transmission facilitated by poor ventilation in the vehicle. A study on the risk of COVID-19 transmission among train passengers in China is presented in Hu (2021). In this study, the authors developed a model that quantifies the risk of transmission among high-speed train passengers on the basis of travel time and distance between passengers. They used data from 2334 COVID-19 patients and the 72,093 close contacts who shared the same journey with them, from 19 December 2019 to 6 March 2020. The results of this study show that the risk varies significantly depending on travel time and spatial distance between passengers. In conclusion, the authors recommend the implementation of measures such as: increasing the distance between passenger seats, reducing passenger density, adequate ventilation and filtration of air, the wearing of face masks and the use of personal hygiene practices. Severo (2021) analyzes the role that the urban rail system in the city of Lisbon has played in the transmission of COVID-19 in said city. The authors analyzed the use of this infrastructure by the inhabitants of the city, together with data on confirmed SARS-CoV-2 infections in the period from 2 March to 5 July 2020 and, using geographic information, linked the cases to the nearest train stations. The authors concluded that there is no consistent relationship between proximity to train stations and the spread of the disease, suggesting that factors such as socioeconomic deprivation are the determinants of infection dynamics.

### Limits of the literature and main contributions

With regard to studies based on inferred contact networks using surveys as a data source, the comprehensive and systematic review by (Hoang 2019) outlines the following considerations. One challenge to be addressed is that of selecting the most suitable sample of people to participate in these studies. It is important that the initial sample is bias-free and representative of the target population. In this type of study, participants typically number around one thousand and data are manually recorded in retrospective surveys. It is therefore necessary to consider the difficulty for participants to recall all the details of the contacts they have had with the required precision. This problem is particularly acute when it comes to recording casual close contacts that do not involve physical contact or verbal exchanges. Furthermore, if the aim is to analyse the variability of contacts within the same population group, participants would need to report on their contacts over a considerable period of time, which is also problematic. Finally, as egocentric sampling is used in this type of study, certain characteristics of the resulting contact network cannot be estimated.

The main limitation of methodologies that infer contact networks through simulation is that their inferences are based on knowledge of how different population groups are spatially structured at different scales (families, villages, cities, metropolitan areas and regions) and what the mobility of people is like at these different scales. Therefore, in this type of methodology, the inferred networks are designed to represent large-scale interactions, providing only very general information about contacts between individuals.

With regard to work based on the use of proximity sensors, one constraint faced by this type of study is the difficulty of deploying the technological infrastructure required to conduct a study involving a large number of people, over a long period of time and covering a wide geographical area. Another problem that arises in this type of work is underrecording due to environmental factors that hinder signal propagation or improper use of the sensors by participants. Conversely, improper use of the sensors by participants can also cause an overrecording problem. Finally, in this type of study, the definition of contact refers only to the distance between the people involved, and it is not possible to record whether there has been a conversation or physical contact.

The proposal made in this paper is a case of a hybrid methodology developed for the analysis of close contacts that occur in the specific context of intercity public road transport systems. It defines close contact in terms of the distance separating the people involved in the contact, as used in methodologies based on proximity contacts. Using data commonly used in transport systems—in which contactless card-based payment systems play a major role—and data mining, it is able to generate the network of close contacts of passengers who have travelled together in the same public transport vehicle. These close contacts are obtained by simulations based on seat allocation strategies. Therefore, it also falls into the category of methodologies that use simulations to infer the network of contacts.

In keeping with its principles of operation, the contacts can be studied in detail, as they have all the required attributes: frequency of contact, duration, location, time of occurrence and relevant information on the persons generating the contacts. As the data from which the contacts are generated are obtained automatically and continuously, it is possible to carry out studies involving a large number of participants, yielding samples with a large number of contacts. Thanks to the processes for validating the integrity of the collected data, the contact information is not only complete, but also reliable. It follows, therefore, that the challenge posed by the methodologies described in the review of related work—that of having a significant sample of close contacts with reliable data and a large number of participants—is solved by the proposed methodology. Also, another common limitation solved by the proposed methodology is that it allows for the continuous study of close contacts for the purpose of analysing their variability. In the specific context of methodologies based on the use of proximity sensors, the proposed methodology, by taking elements used by public transport users on a daily basis, does not require specific technological deployments to conduct the research. Therefore, it enables users across the entire transport network to participate naturally, while also preventing errors in the data due to improper use of the devices used for data acquisition. In the specific context of simulation-based studies, the proposed methodology supports precise studies on individual close contacts between people participating in the study, an aspect that is not addressed by related works in this type of study. Finally, in the context of work on epidemiology in transport systems, the proposed methodology is original in terms of both purpose and design.

Table 1 shows a summary of the relevant characteristics of the methodologies that are generally used and that have been presented and of the results obtained by applying them to obtain contact networks.

**Table 1:** Summary of the relevant characteristics of different methodologies

Work	Population type	Methodology for generating contact network	Contact types	Recorded information (Study period)	Participant sample size (contacts/interactions sample size)
Ferguson 2005	General	Simulation based on instantaneous population density	Co-presence in households, schools and workplaces	NA	USA synthetic population of 300 million UK synthetic population of 58.1 million (NA)
Longini 2005	General	Simulation bases on census data, demographic information and social network data	Co-presence in households, schools and workplaces	NA	NA
Wallinga 2006	General, excluding 0–1 year	Survey (face-to-face interview)	Conversational	Age location (1 week)	2106
Moosong 2008	General	Survey (Self-report)	Physical Conversational	Age location, duration frequency (1 day)	7297 (97,904)
Klepac 2018	General	Survey (Self-report)	Physical conversational	Age location (1 day)	40,177
Meyers 2005	General	Simulation based on census data, demographic information, and social network data	Co-presence in households, schools, workplaces, hospitals and other public places	Simulated location	Vancouver synthetic population of 2600 people
Chao 2010	General	Simulation based on census data, demographic information, and census transportation	Co-presence in households, schools, workplaces and community	Simulated location	USA synthetic population of 280 million
Isella 2011a	Event participants	Sensor proximity (RFID)	Distance proximity	Contact participants duration frequency (3 months)	100 people in Scientific conference (10,000), 14,000 people in museum exhibition (23,000)
Cattuto 2010	Event participants	Sensor proximity (Wireless sensor)	Distance proximity	Contact participants duration frequency (12, 3 and 2 days)	25 people in exhibition (8700), 575 people in Scientific conference (17,000), 405 people in Scientific conference (60,000)
Salathé 2010	School community	Sensor proximity (Wireless sensor)	Distance proximity	Contact participants duration frequency (1 day)	778 (762,868)
Isella 2011b	Paediatric hospital service community	Sensor proximity (RFID)	Distance proximity	Contact participants duration frequency (7 days)	119 (16,000)
Stehlé 2011	School community	Sensor proximity (RFID)	Distance proximity	Contact participants duration frequency (2 days)	242 (77,602)
Stopczynski 2015	University students	Sensor proximity (Bluetooth)	Distance proximity	Contact participants duration frequency (28 days)	464 (1,472,090)
Proposed Methodology	Intercity public transport system users	Sensor Proximity (Contactless smart cards) Data Mining Data driven simulation	Distance proximity	Contact participants duration frequency vehicle (31 days)	43,804 (176,892)

## Methodology

In this section, we present a methodology based on data mining designed to obtain data of epidemiological interest and the patterns they follow from mobility data generated in a road transport system. The data of interest are obtained on the basis of the definition of close contact established by national and international health agencies for COVID-19, which will be presented and formalized in the following section describing the formal model adopted by the methodology. Specifically, in the context of road transport systems, the data of interest relate to the frequency with which regular users of the transport system travel in the same vehicle, and the duration and distance over which these events occur; the study looks at how these aspects vary according to the age of the users. From these data, estimates can be made of the interactions that occur between users of the transport system that can lead to contagion, obtaining the patterns of these interactions according to the age groups of the users. Therefore, and as expressed in the section on related work, the data obtained are of interest for epidemiological models that require data that represent the patterns of contacts between people belonging to different age groups, i.e. studies that try to establish “who infects whom” (Hoang 2019). Implementation of the methodology produces a framework for obtaining this type of information according to epidemiological and transport system parameters.

### Formalization

In a model of the spread of a respiratory infectious disease based on social contact, it is assumed that the number of potentially infectious contacts between two different groups of people is proportional to the number of social contacts between people in those groups, and that this proportion is determined by a proportionality factor, q, which measures the infectivity of the disease, the value of which is disease-specific (Wallinga 2006).

The next generation matrix M’ is a matrix used to estimate how an epidemic will spread based on the contacts that occur between different population groups. Matrix M’ is obtained from contact matrix M. In matrix M, each element m_ij_ represents the average number of people from group i who have been in contact with a person from group j. In general, the reciprocity principle is assumed for these contacts. This means that if c_ij_ represents the total number of contacts between people from group i and people from group j, and c_ji_ is the total number of contacts between people from group j and people from group i, then c_ij_ = c_ji_. If w_i_ is the number of people in group i and w_j_ is the number of people in group j, then m_ij_ = c_ij_/w_j_ and m_ji_ = c_ji_/w_j_. Finally, if q is the proportional infectivity factor of a disease, matrix M’ is obtained by the expression M’ = qM, so n_ij_ = qm_ij,_ where n_ij_ is an element of the M' matrix.

If the age of the people in a population is known, and they are grouped by age intervals, and matrix M resulting from the contacts between people from the different age groups is available, then for an infectious disease with a known infectivity factor q, it is possible to estimate how many people in the different age groups will be infected by a sick person in a given age group. This estimation is performed using matrix M’ and vector X_0_ which represents the number of people who are initially infected in each group. The result of the expression X_i_ = (M’)^i^X_0_ represents the estimated number of people in each age group who will be infected at the i-th iteration of the spread of a disease (Moosong 2008).

Assuming that all age groups are equally susceptible to infection, the relevant property of matrix M’ is that its dominant eigenvalue matches the basic reproduction number R0 mentioned above. The dominant eigenvector of M’ indicates the level of contribution of each age group to the spread of the disease (Diekmann 1990).

In the context of respiratory infectious diseases, and in the specific context of the COVID-19 pandemic, in general and at the community level, a close contact is considered to be any person who has been within 2 m of an infected person for a total cumulative time of 15 min or more over a 24-h period.^3^ Based on this definition and in line with the epidemiological studies described in the previous section, the objective of the proposed methodology is to estimate the number of close interactions between passengers in an intercity road transport system. The term interaction and not contact is used because, according to the above definition of close contact, it entails an interaction with an infected person and no personal passenger data is handled in the proposed methodology. In the formal framework of the methodology, *interaction* is defined as an event consisting of two people being within two meters of each other continuously for a period of time; the duration of the interaction is defined as that period of time. When one or more interactions with a cumulative duration of more than 15 min occur between two people in a 24-h period, then a *close interaction* event occurs between them. Considering the definition of *close interaction*, the objective of the proposed methodology is to obtain useful epidemiological information on interactions between passengers in a road transport system, as well as to provide information on passengers travelling in the same vehicle during an interval of time. This is referred to as co-presence of users.

Table 2 summarizes the notation used in the formalization described below.

**Table 2:** Notation of the formal model used by the methodology

Notation	Meaning
*n*_*i*_	Node on the transport network. Each node is associated with a stop. Subscript *i* is an integer value that uniquely identifies the node
*N*	Set of transport network nodes
*a*_*i*_	Transport network arc. Each arc directly links two nodes of set *N* of the transport network
*A*	Set of arcs on the transport network directly linking two nodes
*G(N,A)*	Directed graph representing the transport network
*r*_*i*_	Route on the transport network. Subscript *i* is an integer value that uniquely identifies the route
*R*	Set of defined routes on the transport network
*J*_*i*_	Vehicle journey on route *i*
*J*	Set of vehicle journeys on all defined routes on the transport network
*T*	Period of time
*t*	Moment of time in period *T*
*J*_*T*_	Set of vehicle journeys that have been completed in time period *T*
*J*_*T,i*_	Set of vehicle journeys on route *i* that have been completed in period *T*
*J*_*t,i*_	Set of vehicle journeys on route *i* that started at moment *t*
*v*	Public transport vehicle
*J*_*t,i,v*_	Vehicle journey on route *i* that started at moment *t* carried out by vehicle *v*

Graph theory has been used for different purposes and with success in studies in the field of intelligent transport systems (Aleksander 2020; Henning 2017). By modelling the road network as a spatial graph, it is possible to formalise the routes that public transport vehicles can take. From this physical representation of the transport network, other more conceptual graphs associated with transport activity are constructed at higher levels. In addition, formal mathematical graph theory can be applied to solve common problems in transport operations, such as optimal route design, both from the point of view of distance and estimated travel times, operating costs and detection of bottlenecks in the transport network (Qu 2019).

Thus, the transport network was formalised on the first level of the formal framework by means of a graph, G:

$${\text{G }} = {\text{ G}}\left( {{\text{N}},{\text{A}}} \right)$$

where:

N represents the set of nodes in the transport network where passengers board or alight from vehicles, N = {ni}, and subscript i is the point identifier.

A represents the set of arcs linking two nodes, A = {ai}, where subscript i is the arc identifier.

Each arc represents the direct path taken by a public transport vehicle carrying passengers between an origin node and a destination node. Therefore, *G* is a directed graph. The next entity that is formalized at this level is the concept of a route. A route is defined as the path taken by vehicles carrying passengers. Considering graph *G*, a route is defined as an ordered sequence of arcs (*a*_*i*_, …, *a*_*n*_), where *a*_*i*_ … *a*_*n*_ ∈ *A*. The set of routes taken in the transport network is represented by *R*, *R* = *{r*_*i*_*}*, where subscript *i* is the route identifier. A route segment *r*_*i*_ is defined as an ordered sequence of arcs (*a*_*p*_, …, *a*_*q*_) included in route *r*_*i*_.

For the purposes of the methodology, the type of operation that is of interest is the completion of a route by a vehicle to transport passengers, called a *vehicle journey*. In the proposed notation, the set of vehicle journeys that are completed in the transport network is represented by *J*, *J* = *{J*_*i*_*}. J*_*i*_ represents the set of vehicle journeys that consist of transporting passengers on the route identified by subscript *i*. Alternatively, the set of vehicle journeys, irrespective of the route followed, that are completed in time period *T* is represented by the notation *J*_*T*_. The set of vehicle journeys consisting of transporting passengers on route *i* during time period *T* is represented by *J*_*T,i*_. If, instead of time period *T*, we have moment of time *t*, then *J*_*t,i*_ represents the set of vehicle journeys on route *i* for which the start time is *t*. Finally, if *v* identifies a vehicle, then *j*_*t,i,v*_ represents a vehicle journey on route *i*, which begins at time *t* and which is performed by vehicle *v*. The trip taken by a passenger on vehicle journey *j*_*t,i,v*_ is defined as the route segment *(a*_*p*_*, …, a*_*q*_*)* that the vehicle has travelled while the passenger is in the vehicle. The duration of the trip the passenger has made is the time elapsed since the passenger boards the vehicle at the origin node of directed arc *a*_*p*_ and alights at the destination node of directed arc *a*_*q*_.

Finally, to define the type of event in the vehicles, the concepts of *co-presence* event, *interaction* event and *close interaction* event between two users, *p*_*1*_ and *p*_*2*_, of the transport system were formalized according to whether the following three conditions are fulfilled:

- C1. Both have travelled on the same vehicle journey, *j*_*t,i,v*_.
- C2. The trips made by *p*_*1*_ and *p*_*2*_ on *j*_*t,i,v*_ have at least one common arc.
- C3. Passengers *p*_*1*_ and *p*_*2*_ have been less than 2 m apart during the path of the common arcs of the trips of *p*_*1*_ and *p*_*2*_ in *j*_*t,i,v*_.

And from these, the events are defined as.

- *Co-presence* event between *p*_*1*_ and *p*_*2*_: if conditions C1 and C2 are met.
- *Interaction* event between *p*_*1*_ and *p*_*2*_: if conditions C1, C2 and C3 are met.
- *Close interaction* event: if one or more interaction events occur within a 24-h interval between passengers *p*_*1*_ and *p*_*2*_ and the cumulative duration of these interactions exceeds 15 min.

Formally, in an environment in which co-presence interactions and close interactions occur, as is the case of public transport vehicles in this formal model, the co-presence interaction network and the close interaction network have some common structural and statistical features, the former being much denser than the latter (Génois 2018).

Given the definition of the concepts of co-presence interaction and close interaction, in an epidemic scenario where the main form of transmission is through close contacts, the number of close interactions that occur is an indicator of infection risk in the transport system. In the case where aerosols are the primary mode of transmission, the number of co-presence interactions is an indicator of infection risk. It is therefore of interest to have an environment in which this number can be estimated and to assess how this number is affected by various measures that transport operators can take to reduce it.

**Fig. 1:**
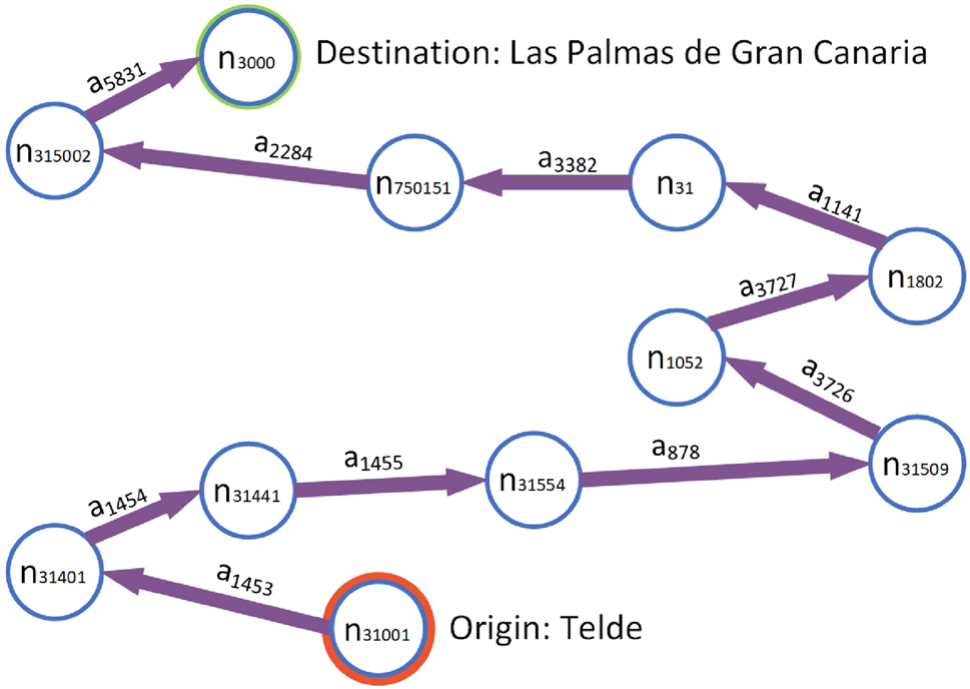
Example of the proposed notation on a route

Figure 1 illustrates the application of this notation to an example route and the vehicle journeys along it.

This example is a real case of a route on the transport system selected as a use case for the methodology, which will be described later on. The identifier code of the route is 534, represented by *r*_*534*_, it has 11 stops, and each stop is represented by a node whose subscript is its numerical identifier. These nodes are a subset of set *N* which represents the nodes of the transport network. The 10 directed arcs linking each pair of nodes are represented by purple arrows and the subscript of each arc corresponds to its numerical identifier. These arcs form a subset of set *A* which represents the arcs of the transport network. All vehicle journeys along route *r*_*534*_ are represented as *J*_*534*_. Taking as an example time period *T* consisting of the whole month of December 2019, the vehicle journeys along this route were planned to run from Monday to Friday, excluding public holidays, every 20 min from 11:10 to 16:30, and every 30 min from 16:55 to 20:55. Therefore, for this type of day, 26 journeys per day were scheduled. Vehicle journeys were also scheduled on non-holiday Saturdays every 30 min from 06:25 to 12:25. Therefore, for this type of day, 15 journeys per day were scheduled. The set of vehicle journeys on route *r*_*534*_ in period *T*, represented by *J*_*T,534*_, consists of all the journeys completed according to this schedule. One element of set *J*_*T,534*_ is the vehicle journey that started on Monday 2 December 2019 at 11:10. Assuming that *t* represents the date and time indicated and that the vehicle that completed the journey is identified by code 1028, then this journey would be represented by *J*_*t,534,1028*_.

### Considerations on intercity transport systems: challenges to be addressed

With the commonly used technologies of intercity road transport systems, especially automatic vehicle location systems and automatic payment systems, it is possible to obtain information about the trip made by passengers: at which node they started their trip, which vehicle they used and at what time they boarded the vehicle. However, it is not always as straightforward to identify the end point and the duration of a passenger’s trip, as there is often no record of the time at which the passenger alights from the vehicle. Nevertheless, it is possible to determine the destinations of regular users of the transport system by mining the data related to the trips they make, since these users generally use smart payment systems, such as contactless cards, which automatically record the transactions made in payment for the trips they make. Estimating the destination stop, for trips where this information is not available, is a challenge for the methodology and is explained in Sect. 3.2.1.

With regard to the distance between passengers, in the case of urban road transport systems, passengers may travel standing and seated, and move within the vehicle without any impediment, so it is not possible to determine this variable using these technologies alone. However, in the case of intercity road transport systems, passengers are generally not allowed to stand when travelling, and only in certain circumstances and on short journeys, since the vehicles used in such systems are not designed for standing passengers. However, it is not always possible to determine the distance between passengers during a vehicle journey, since in intercity transport systems that are not long-distance, passengers are not assigned a seat at the time of travel and therefore, when they enter a vehicle, they can occupy the free seat of their choice. The seat occupied by each passenger on their trip is not known, so estimation of this seat will be the second challenge faced by the methodology. Section 3.2.2 explains how this challenge has been addressed in the methodology.

#### Addressing values missing from the transport system data: destination stop estimation

In data mining projects, the challenge of how to handle datasets with missing values often has to be addressed. In a general context, Dinh (2021) proposed a novel method, called Clustering Mixed Numerical and Categorical Data with Missing Values (*k*-CMM), to classify datasets with a high number of missing values. In the specific context of traffic accident data analysis, this challenge was tackled by Deb (2016), who proposed a method based on decision trees. As mentioned above, information is not always available on the destination of passengers and the time at which they alighted from the vehicle, which are essential to ascertain their journey time and to identify their interactions. However, there are two types of users whose trips have a very definite pattern in terms of the destination stops:

- Passengers that make multi-stage trips, such that the end node of one stage (transfer node) is close to the start node of the next stage.
- Passengers who make single-stage trips to their place of work, study, public service or leisure and who also return using the transport system.

The trips made by this type of passenger exhibit a common pattern: on two consecutive journeys made by the same passenger, the destination node of the first is located within a short distance of the origin node of the second. This proximity is determined by a distance threshold that depends on the type of transport network, smaller in the case of urban transport and larger in the case of intercity transport, U_p_. However, to distinguish this case from any other that may occur on the user’s journey, it is necessary to introduce a second distance threshold, which determines whether two nodes of the network, without being the same, are similar for the purpose of estimating the destination of the first journey, U_s_. This is the case for those trips that start from the same geographical location but with a different destination (at intersections or on both sides of a two-way road), or from two consecutive nodes on the same route, but very close to each other.

In line with the techniques proposed by (Li 2011) and (He 2015), a procedure was developed to infer the final destination of the trips made by passenger *p* – from one of the two categories above – when this information has not been recorded. This procedure is based on the available data on trips made by *p* on the different vehicle journeys *J*_*t,i,v*_. For each of these vehicle journeys, node *n*, at which *p* started the trip, and time *t’* of the start of the trip, are known. Node *n* is the origin node of one of the arcs that form a sequence of arcs *(a*_*p*_*, …., a*_*q*_*)* that form the segment of route *i* travelled in *J*_*t,i,v*_. Moreover, *t* ≤ *t’*, meaning that the start of the user’s trip, *t’*, is equal to or later than the start of vehicle journey *t*. The purpose of the procedure is to ascertain the final stop of the trip made by *p* on *J*_*i,t,v*_ and, therefore, the sequence of arcs that form the segment of route *i* travelled by *p*. To deduce the final stop *q* of journey *J*_*t1,i1,v1*_, the procedure uses the known data for the next journey made by *p*. If *J*_*t2,i2,v2*_ is the next journey made by *p*, then node *n*_*2*_ and time *t”* at which he/she started the journey are known. If nodes *n*_*1*_ and *n*_*2*_, the starting nodes of the two vehicle journeys, are not considered as similar, that is, the Euclidean distance between both is greater than the U_s_ threshold mentioned above, the final stop *q* of the trip made by *p* on *J*_*t1,i1,v1*_ would be the stop on route *i*_*1*_ closest to stop *n*_*2*_ at which *p* started the trip on *J*_*t2,i2,v2*_*,* provided that this final node *q* is located at a distance from *n*_*2*_ that does not exceed the U_p_ proximity threshold indicated above, that is, it is not too far away. Once the final stop has been deduced, the time of the trip made by *p* will be the sum of the time taken by *v* to traverse the sequence of arcs *(a*_*n1*_*, …, a*_*q*_*)*.

Taking the notation used in the description of this process, Algorithm 1 provides an estimation of the destination stop of the trip made by passenger p in vehicle journey *J*_*t1,i1,v1*_.
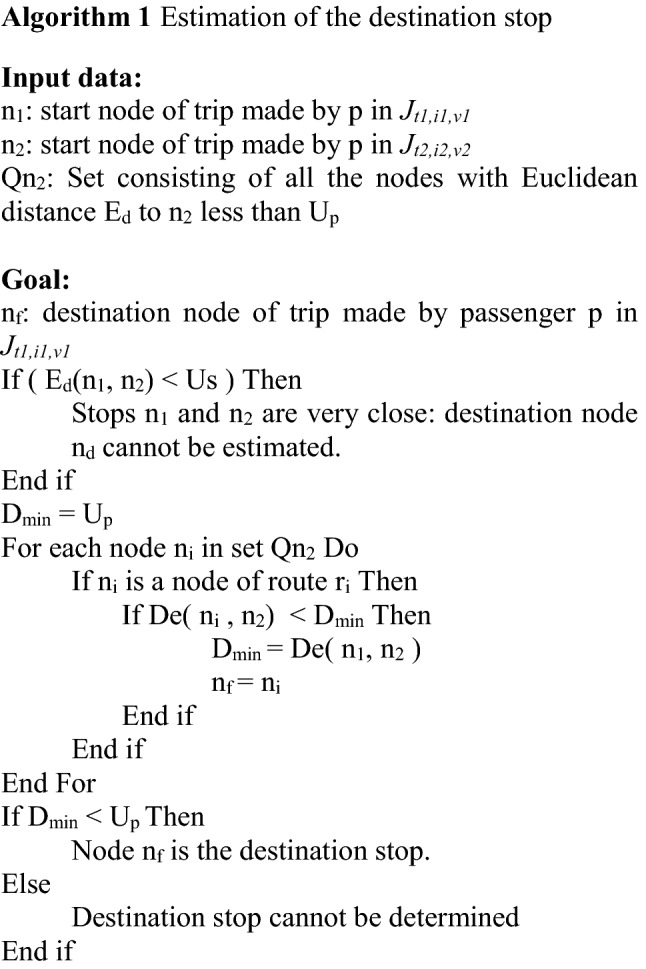


#### Estimation of unknown data: estimation of the seat occupied by a passenger

This section describes how to estimate the seat occupied by a passenger during their trip. If it is assumed that passengers can choose any free seat they wish, then the objective of this estimation is to approximate the passenger’s behaviour in making this choice as closely as possible. In the case where the estimation is based on an objective to be achieved, such as minimising infection risk for passengers travelling on a vehicle journey, then a complex problem arises in which there are multiple individual objectives and an overall objective that is defined a priori. The individual objective is to minimise the close interactions of each passenger travelling on the vehicle journey, which depends on the seats occupied by the passengers in the vehicle at the time the passenger enters and the seats that will be occupied by subsequent passengers. The overall objective defined a priori is to minimise the number of total interactions. In this context of complex decision-making problems, hybrid methodologies using multi-criteria programming and data clustering (Petchrompo 2021) or combinations of mathematical models such as DEMATEL (Decision Making Trial and Evaluation Laboratory) and PROMETHEE (Preference Ranking Organisation Method for Enrichment Evaluation) (Pegoraro 2021) have recently been proposed. Also in the context of complex problems affecting manufacturing or production processes, hybrid methodologies combining machine learning, big data and simulation techniques have recently been proposed (Gao 2022).

**Fig. 2:**
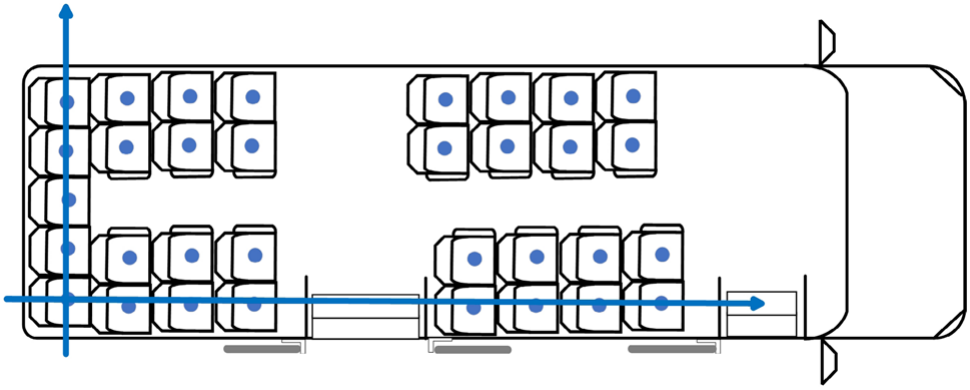
Schematic representation of vehicle

Considering the observations made on intercity road transport systems, the assumption that all passengers are seated is realistic. In the methodology, the distance between two passengers travelling in a vehicle is defined as the distance between the center points of the seats they occupy. In order to systematize the process of obtaining the seat center points and thus automatically obtain the distances between seats, a two-dimensional representation model of the vehicle space for passengers was developed that takes into account the wide variety of bodywork types used in intercity transport systems. This spatial representation is based on the vehicle bodywork drawings and measurements taken on the vehicles. Figure 2 shows a representation of the passenger areas of the two types of vehicles considered in this study.

This paper assumes that the passenger remains in the same seat for the entire trip, and two seat allocation policies are considered:

- Empirical policy (EP). This policy is based on observed behavior whereby the user prefers not to sit next to another passenger, without any other consideration.
- Minimize risk policy (MRP). This policy consists of assigning the user to the free seat that is more than 2 m away from the largest number of passengers, in order to avoid as many interactions as possible with passengers on board the vehicle when boarding.

The EP policy, which approximates passenger behaviour, is used as a baseline to compare the effectiveness of the MRP strategy. As stated, the EP policy does not take into account the fact that passengers may travel together and therefore be seated as close together as possible in the vehicle. Nor does it take into account the fact that there may be passengers who do not mind sitting next to another passenger, when other seats are further away. For this reason, it is reasonable to assume that the close interactions that occur in reality would be greater than those that occur when applying the EP strategy. Therefore, the effect of the MRP policy, in terms of reducing the number of close interactions, would be greater than the interactions that occur in a real case.

If the occupancy of the vehicle does not permit strict application of the allocation criterion, then a seat is randomly allocated from the vacant seats that are in the best circumstances according to the allocation policy used.

The allocation procedure is based on three parameters the values of which vary according to the allocation policy. These parameters are:

- *Safety distance*. In the case of EP, this is the minimum distance between the centers of two adjacent seats, and in the case of MRP, it is 2 m.
- *Affected seats list*. This is a list for each seat in each bodywork type in the fleet, showing the number of seats that are affected by occupancy of the seat. This list depends directly on the value of the *safety distance* parameter as determined by the allocation policy used.
- *Potential risk* of a seat. This is a value that is assigned to each of the free seats in the vehicle during the course of a vehicle journey. The value increases as the seats that appear in the *affected seats list* are occupied and decreases when any of these seats are vacated.

The procedure simulates seat occupancy by passengers in each vehicle journey *J*_*t,i,v*_, taking as input parameters the affected seat list pertaining to the vehicle bodywork type and the safety distance of the policy to be applied and, as input data, each of the trips made by passengers on that vehicle journey (see Algorithm 2).
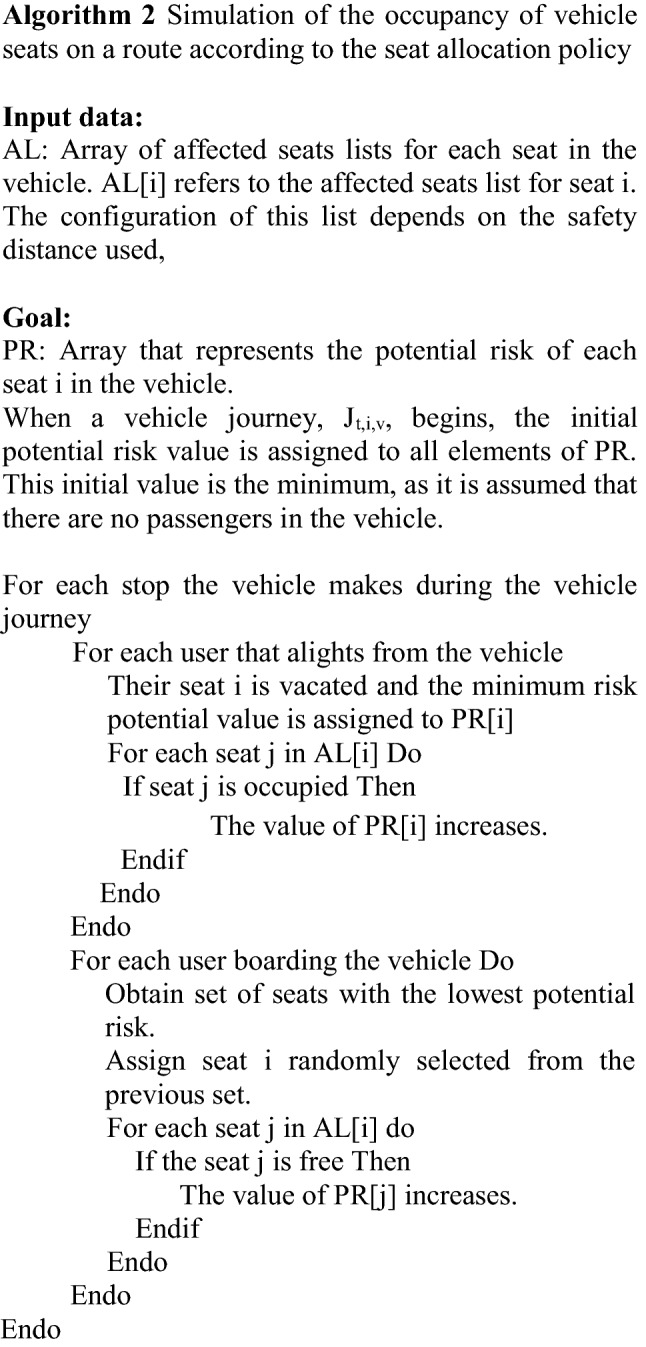


### Data model

Based on the above, the proposed data model is divided into two categories. The first, named Graph Database, represents the entities and relationships that correspond to levels 1 and 2 of the described formalization of the transport system: the network of stops, the definition of the route services, and the operations and trips made on them. The second level represents the different events covered by the methodology and those related to seat allocation, which are not found in the initial database.

**Fig. 3:**
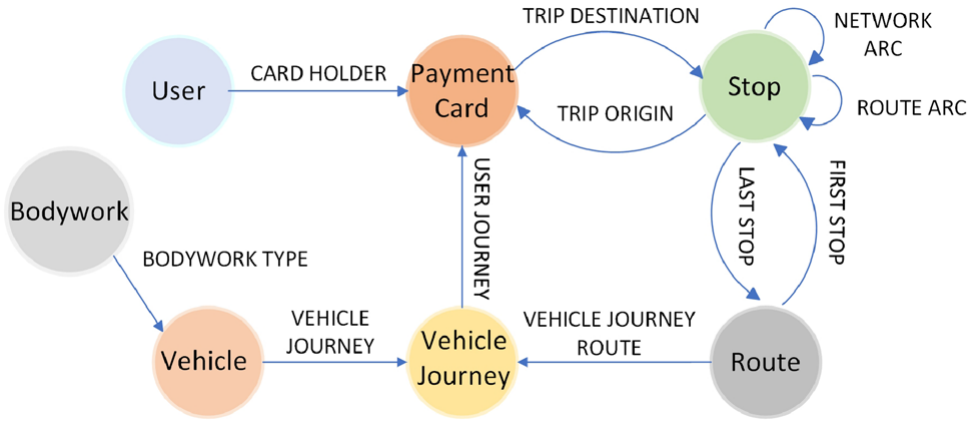
Schema of the Graph Database

In the case of the Graph Database, choosing a suitable database structure, which reflects the entities of interest and their relationships in a meaningful and robust way, without the need for repeated inferences, is of particular importance when dealing with a large volume of data. This is why we have opted for a graph database – making it easier to obtain data on the concurrence of users in vehicles (the main object of analysis of this work) – called a transport system graph (TSG). The schema is presented in Fig. 3, which illustrates the graph representing the entities involved and the relationships between them.

In the network arc relationship, the travel time attribute indicates the estimated time it takes for a vehicle to travel from the origin stop of the arc to the destination stop. In the first stop and end stop relationships, the time attribute specifies at what time the user boarded or alighted from the vehicle on the vehicle journey.

The Events and Seats category represents co-presence and interaction events. From this category, different datasets will be generated for each type of event to be analyzed: set of participating users, duration of journeys and times at which they take place, and event matrices, inter alia. The following data structure is proposed to represent the two types of events (Table 3). This structure contains the following fields: event start date and time, passenger p1 user key, passenger p1 age group, passenger p2 user key, passenger p2 age group, number of events, total event duration.

**Table 3:** Event data structure

Start date and time	p1 user key	p1 age group	p2 user key	p2 age group	Number of events	Total duration

While co-presence events may be obtained directly from the graph defined in the transport system category described above, for the estimation of interaction and close interaction events, information on the distribution of the seats in the vehicle in which the passenger made the trip is required to, on the one hand, simulate the user’s choice of seat for the trip and, on the other hand, determine the passengers who are less than 2 m away from him/her. For this purpose, an additional data structure has been developed that represents, for each bodywork type in the fleet and distance *d*, *b*_*td*_, the list, for each seat *a*_*i*_, of all the seats {*a*_*j,*_* a*_*k, …,*_* a*_*n*_} that are at a distance of less than *d* (the *list of affected seats* described above). Schematically, this structure is shown in Table 4.

### Implementation

**Fig. 4:**
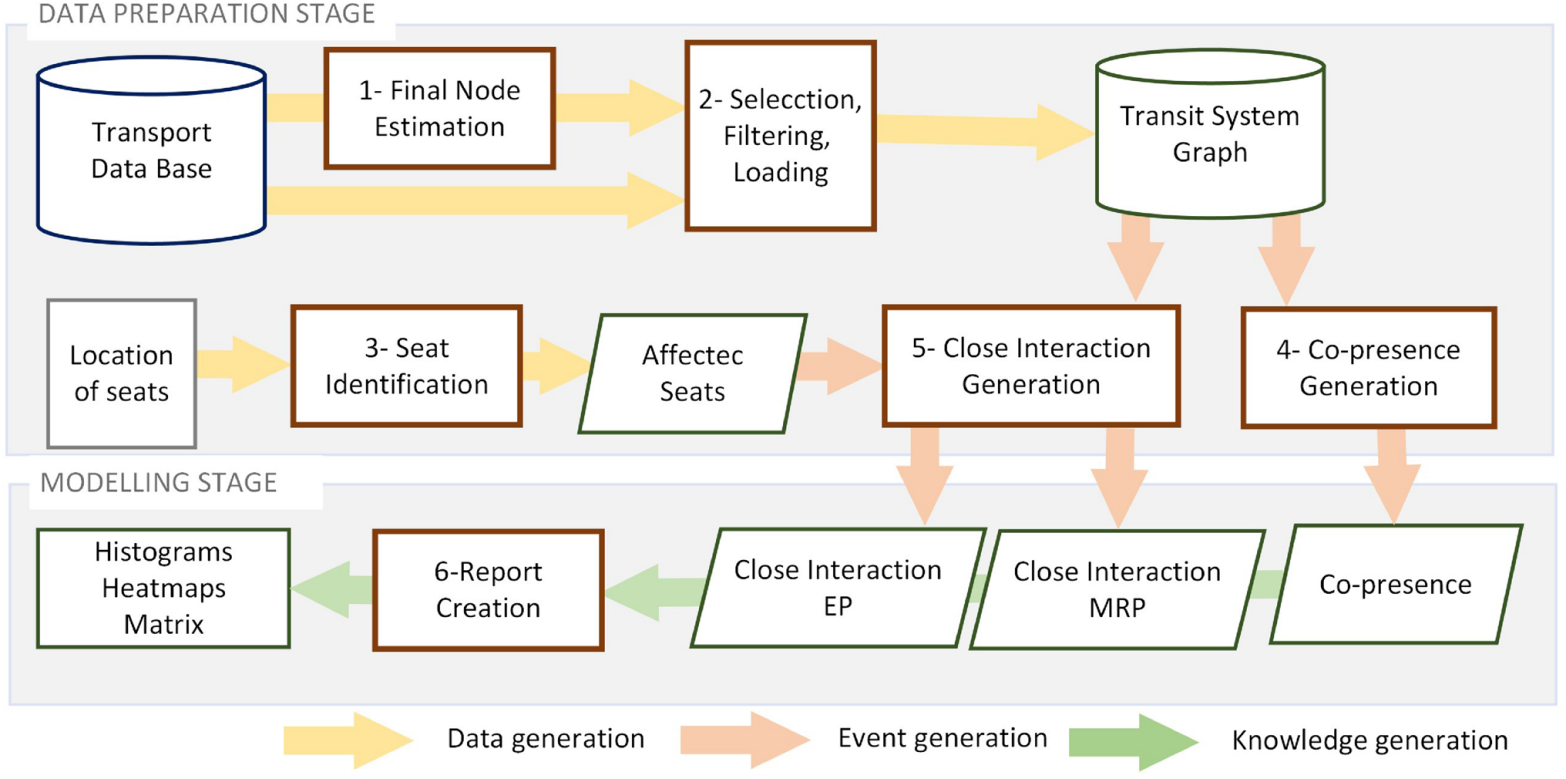
General process diagram

The processes and data involved in implementing the proposed methodology are schematically presented in Fig. 4. It is structured in two stages: the data preparation stage and the modelling stage. These stages are described below along with some concepts that will be used later in the Use Case section.

The purpose of the data preparation stage is to generate the co-presence and close interaction events defined in the formalization of the problem. These events are the starting point of the modelling stage. In this stage, three processes are executed sequentially to produce the data required for generating the co-presence and close interaction events. The first process – final node estimation – estimates the final node of user journeys. The second – selection, filtering and loading – encompasses all the tasks related to the generation and loading of the TSG from, on the one hand, the records contained in the TDB relating to the transport network, vehicles, users, contactless cards, services and trips made, and on the other, the destination stops as estimated by the previous procedure, guaranteeing the reliability, accuracy, completeness and consistency of all the data. The third – seat identification – generates the list of affected seats described in Sect. 3.2.2 for each bodywork type in the fleet and for each safety distance considered, based on the two-dimensional representation of the seats. Once the data is generated, the event generation processes are executed. The co-presence events are generated through the co-presence process and the close interaction events through processes that are implemented according to epidemiological parameters (safety distance and cumulative duration of the interactions) and the seat allocation strategy. In this case two have been implemented: the close interaction EP process for the EP strategy and the close interaction MRP process for the MRP strategy.

The generation of close interactions is a key step in the methodology. In the case of the road transport systems for which this methodology has been developed – those in which passengers are not assigned a seat in the vehicle – the distance between passengers who travel on the same route segment in the same vehicle journey is not known. To obtain this information, the methodology first uses the procedure for estimating the destination stop on those journeys where this information is not available, as described in Sect. 3.2.1, and, once the origins and destinations of the journeys are known, the procedure that performs the “simulated” assignment of a seat to a passenger on his or her journey is performed, making use of two alternative seat assignment policies, as described in Sect. 3.2.2. To go into more detail, given that for all passengers travelling on the same vehicle journey, the origin stop and the destination stop of their trip are known (destination stop estimation process) and all have been assigned a seat, according to the seat assignment policy applied, then all the interaction events that occur between passengers on the vehicle journey can be obtained. These events are recorded in a data structure described in Table 3. The passengers involved in each interaction are retrieved from the identifier of the payment card used by the passenger, as this identifier is unique for each card. The distance between passengers is the distance between the seats occupied by each passenger. The shared route segment on which both passengers involved in the interaction coincide is obtained from the origin and destination stops of the trip made by each of the two passengers. The duration of the interaction is the time spent by the vehicle in travelling the shared route segment and this time is the sum of the travel times of the arcs *a*_*i*_ joining the sequence of stops that are part of the shared segment travelled by the two passengers. All interactions between passengers that coincide on a shared route segment in the same vehicle journey are of the co-presence type. To obtain the close interactions that occur between two passengers, as described in Sect. 3.1 (where the concept of close interaction was defined), the first step is to obtain their co-presence interactions that have occurred in a 24-h window, and the second step is to select those whose distances are less than the safety distance of 2 m, using the data records represented in Table 4, and finally, the cumulative duration of these selected interactions is calculated. If the cumulative duration is equal to or greater than 15 min, then a close interaction between the two passengers is considered to have occurred.

**Table 4:** Bodywork type data structure

Bodywork type key	Seat identifier	*Affected seats list*
*b*_*td*_	*a*_*i*_	{*a*_*j,*_* a*_*k, …,*_* a*_*n*_}

The modelling stage includes the tasks related to the production of new information from the sets of events generated, which are included in process 6-Report Creation. In this process, different sets of data are generated (population of participating users, groupings of events according to age of the users, etc.) and different modelling techniques are applied according to the objectives and specific needs to be addressed, which may pertain to the field of statistics or machine learning. In the use case presented below. Considering the data used in epidemiological work on contacts, data of epidemiological interest include the average time of trips made by users from different age groups, the average number of trips made by users from different age groups, the number of interaction events, the number of events that lead to each interaction, the average duration of the events and the co-presence and close interaction matrices. Since no baseline datasets are available to validate the results, the consistency between the different results was analysed.

## Use case

The proposed methodology was applied to the intercity road transport system on the island of Gran Canaria (Canary Islands, Spain). This transport system is operated by the company Global Salcai-Utinsa, which annually transports around 20,000,000 passengers and covers 25,000,000 km. The time period studied was the month of December 2019, two months before the COVID-19 pandemic was declared. The decision to select this month was made because in this period demand was not affected by the travel restrictions imposed by the health authorities as a result of the national health emergency and, in addition, it is a month in which mobility needs fluctuate considerably due to the fact that in this month there are working days, public holidays, school periods, and non-school periods during the Christmas holidays.

To apply the methodology in this use case, the following tools were used: relational database, with the relevant data required for this study from the operator’s transport database, Neo4j to implement the graph database used by the methodology, and the RStudio development environment, for programming the procedures for debugging, completeness, data loading, generation of the different data sets to be analyzed, and the procedures for modelling and obtaining information.

The first step was the evaluation of the final node estimation process. This was applied to trips made with a type of card where users record their exit from the vehicle, using the Euclidean distance E_d_ between the known destination stop and the stop estimated by the procedure as the evaluation metric. This set of trips, for which the destination stop is known, was obtained from the public transport system that was used as a use case for this methodology; the period for which these trips were made matches the period chosen for the methodology, the results of which will be presented in Sect. 4. In the process, the following results were obtained: out of a total of 278 694 user trips, in 26.7% it was not possible to estimate a destination node for the following trip (if any), and in 205 183 (73.6%) this was possible. Table 5 shows the number of trips for which it was possible to estimate the destination stop as a function of the distance between the known destination stop and the estimated stop. The Percentage of cases column shows the percentage of trips relative to the number of trips for which it was possible to estimate the destination stop, where the estimated destination stop is within the distance indicated in the Distance column.

**Table 5:** Distance between the estimated destination node and the actual destination node

Distance	Number of trips	Percentage of cases %
*D* = 0 (actual stop = estimated stop)	98 926	48.21
0 < *d* < 0.5 km	20 630	10.05
0.5 km < = *d* < 0.75 km	21 939	10.69
0.75 km < = *d* < 1 km	5452	2.65
1 km < = *d* < 3 km	17 485	8.52
3 km < = *d*	40 751	19.86

As can be seen in Table 5, 48.21% of the estimated destination stops coincide exactly with the destination stop of the trip made by the passenger, and 71.6% of the estimated stops are located at a distance of less than 1 km and 80.12% at a distance of less than 3 km. For the purposes of the methodology, the travel time of the passenger is a key factor, as this time will be the upper limit of the duration of the interactions between this passenger and the other passengers with whom he/she shares the vehicle. For this reason, to ensure that the estimated travel times do not differ significantly from their actual values, the value adopted for threshold U_p_ in Algorithm 1 is 1 km.

After applying the procedures to select, debug and complete the initial data – obtained from the transport database (TDB) of the aforementioned company – the total number of Graph Database nodes is shown in Table 6.

**Table 6:** Number of nodes (entities) of each type

Node type	Number of entities
Bodywork type	23
User	43,804
Vehicle journey	70,732
Route	440
Stop	2923
Payment card	44,372
Vehicle	443

The number of trips completed in the period considered in this study, using payment cards as a means of payment, is 996184. Of these trips, the destination was known for 60,545 and was not known for 935,639. By applying the destination stop estimation procedure to these 935,639 trips, the destination stop could be estimated for 725,145 trips and could not be estimated for 210,494 trips. As a result, 785,718 of the trips completed in the selected period were represented in the Graph Database, that is, 78.87% of the trips completed in this period. This is therefore a significant percentage of the trips completed in the selected study period. Table 7 shows these results.

**Table 7:** Trips completed in the selected period

Total trips	996,184
Trips with known destination	60,545
Trips with unknown destination	935,639
Trips for which the destination could be estimated	725,145
Trips for which the destination could not be estimated	210,494
Total trips entered in the Graph Database	785,718

### Preliminary data analysis

**Fig 5:**
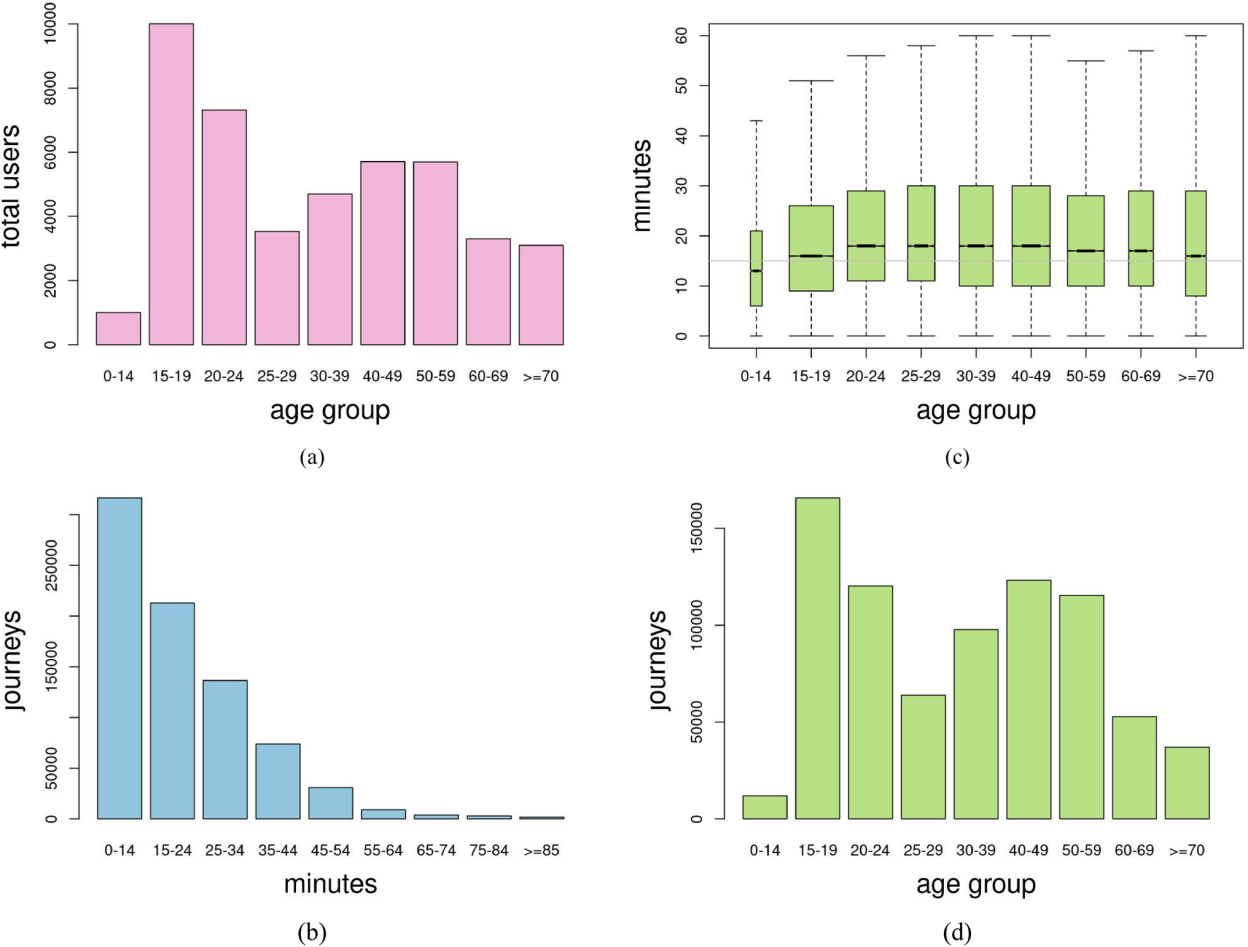
Preliminary values obtained per age group in the period analyzed; **a** Number of users per age group, **b** Number of journeys per time interval, **c** Quartiles of the travel time per age group, and **d** Number of journeys per age group

In a first analysis, information could already be obtained on certain mobility features of the transport system used as a case study. The distribution of the total number of users according to their age is shown in Fig. 5(a). Nine age groups were defined – 0 to 14, 15 to 19, 20 to 24, 25 to 19, 30 to 39, 40 to 49, 50 to 59, 60 to 69, and 70 and over – in order to keep the study population specific, especially in the 15 to 24 age group, which accounts for 46% of the users. Travel times are shown in Fig. 5(b), which shows that approximately 40% of the trips lasted less than 15 min. This is relevant since this is the minimum time for close interaction to take place. Figure 5(c) shows in more detail how travel times are distributed, in this case distinguishing the quartiles for each of the age groups. The graph shows that in the younger age groups, travel times are slightly shorter. The total number of trips made by each group is shown in Fig. 5(d).

Table 8 shows the average number of trips that each user, in the different age groups, made in the chosen study period. This information is relevant because the higher the frequency of trips made, the higher the probability of being involved in the close interactions that occur. It is significant to note that the largest groups of users and those with the most trips—passengers in the 15–19 and 10–24 age groups (see Fig. 5(a, d)—are not the ones who on average have travelled the most in the period under study. The three groups of passengers who, on average, made the most trips were, in that order, the 40–49, 30–39 and 50–59 age groups.

**Table 8:** Average number of trips made by members of each age group in the study period

Age group	Average number of trips made
0–14	11.8
15–19	16.6
20–24	16.4
25–29	18.1
30–39	20.8
40–49	21.6
50–59	20.2
60–69	15.9
> = 70	11.9

### General characteristics of the events

In line with the contact-based studies on epidemiology, this study focused on three key aspects: the number, frequency and duration of interactions on each day of the period analyzed. The close interaction data obtained are the result of applying the EP and MRP allocation policies. It should be noted that, when dealing with close interaction events, where a 24 h window is considered, if more than one interaction occurs in the window, all are associated with the time at which the first interaction occurs.

The number of events is summarized in Table 9, with the median obtained for each of them in the period. It may be concluded that around 12% of the user pairs that travel together in a vehicle at the same time could give rise to a close interaction EP event, and 6% to a close interaction MRP event. In the case of two or more events in 24 h, the percentage drops to 9% for close interaction EP and less than 2% for close interaction MRP. The median durations are summarized in Table 10. In this case, the shorter duration of co-presence has to do with the fact that for close interactions, events lasting less than 15 min are ruled out.

**Table 9:** Summary of median total events

	1 event	2 events	> 2 events
Co-presence	176,892	2897	225
Close interaction EP	20,941	270	13
Close interaction MRP	10,465	56	0

**Table 10:** Summary of median event duration in minutes

	1 event	2 events	> 2 events
Co-presence	14	33	57
Close interaction EP	24	39	58
Close interaction MRP	24	40	59

### Event matrices

In accordance with the definition of the contact matrix described in Sect. 3.1. the event matrices of co-presence and close interaction between the defined age groups of transport system users are presented below. The close interaction matrix was obtained for each of the seat assignment policies. Specifically, the following matrices were obtained:

- Total events matrix (TM). Where each element *t*_*ij*_ represents the total number of events between age groups *i* and *j*. Since the events were generated from users travelling at the same time in the same vehicle journey, *t*_*ij*_ = *t*_*ji*_, this matrix is therefore symmetrical.
- Event matrix for the number of users belonging to each age group (RM). In this case, each element *r*_*ij*_ of this matrix contains the average number of events between transport users of age group *j* with users of age group *i*. Therefore, if *n*_*j*_ is the number of users of age group *j*, and *t*_*ij*_ is the total number of interactions between users from age groups *i* and *j*, then *r*_*ij*_ = *t*_*ij*_*/n*_*j*_. This matrix corresponds to contact matrix M described in Sect. 3.1, for co-presence interactions or close interactions, as appropriate.

**Fig. 6:**
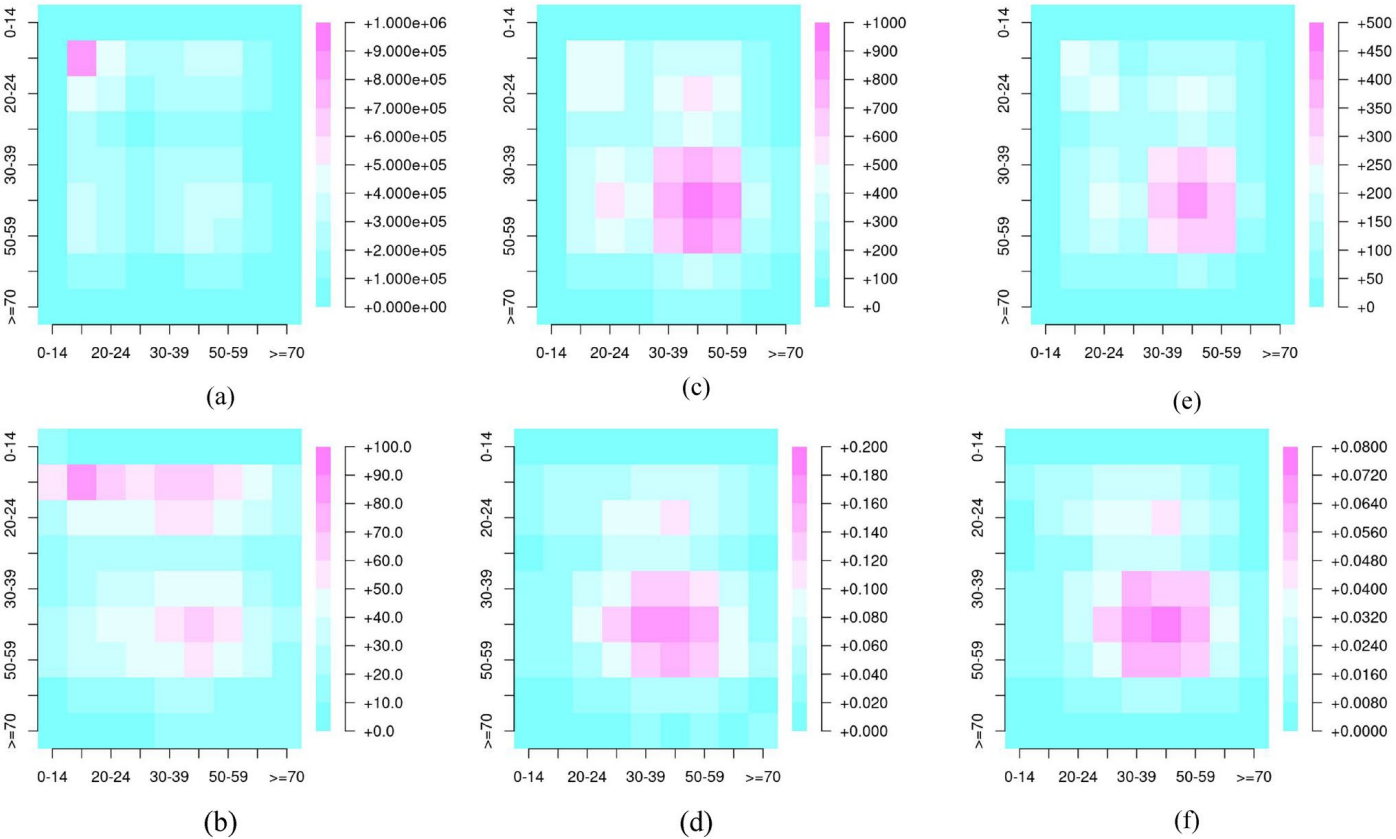
Event matrices; **a** Co-presence interactions TM, **b** Co-presence interactions RM, **c** Close interactions TM applying EP, **d** Close interactions RM, **e** Close interactions TM applying MRP, and **f** Close interactions RM applying MRP

Considering the definition of the events, co-presence is the most numerous set. During the study period, more than 12 million events were generated, which are distributed among the different age groups as shown in Fig. 6(a). In this matrix, the contacts of 15–19-year-olds with each other stand out in particular, with about 1 million occurrences in the period. This group also stands out in the relative event matrix, as can be seen in Fig. 6(b), with slightly more than 100 events with users of the same age group, and where almost all other groups up to the age of 59 also coincide with this group with more than 50 events per user.

A subset of the total number of recorded co-presences can be treated as close interactions if the EP seat allocation policy is applied. For this type of event and allocation policy, the estimated close interactions mostly involve users aged between 30 and 59 years (see Fig. 6(c)). The same age group is also prominent in Fig. 6(d), which represents the RM matrix.

In the case of close interactions applying MRP, the resulting matrices represent a smaller set of interactions compared to EP, because seat allocation under MRP avoids close interactions as much as possible. The overall behavior of interactions between age groups is similar to that observed in the matrices obtained by applying EP, as can be seen in the heatmaps in Fig. 6(e and f), and the number of interactions is more than halved.

### Discussion

This discussion section will focus on results that are epidemiologically relevant and on the complexity of the proposed methodology. Firstly, it will look at results that provide information on the frequency and duration of events in the transport system, and secondly, results that provide information about usage patterns and who interacts with whom in each of the scenarios considered.

A first approximation of duration and frequency was obtained in the preliminary study presented in Sect. 4.1. It should first be mentioned that a considerable percentage of trips made by transport system users have a duration of less than 15 min (Fig. 5(b)). An analysis of journey lengths by age group (Fig. 5(c)) shows that, with the exception of those aged 0–14 years, the median travel time is above 15 min and below 20 min, but the largest age group, 15–19-year-olds (Fig. 5(a)), which also accounts for the largest number of trips (Fig. 5(d)), shows a median time of 16 min, and 40% of trips are shorter than said threshold. The results show that, in the two younger age groups, travel times are slightly shorter, probably due to the importance for these age groups of travelling to their place of study, which is usually not very far from their place of residence. It should be remembered that, as stated in Sect. 3.1, for close contact to occur between two people, they must be less than two meters apart for at least 15 min in a 24-h period.

A more precise analysis was performed in Sect. 4.2, in which the total number of events and their duration were studied on each day of the period analyzed, making a distinction between one, two or more than two events between two users. Table 9 summarizes each case with the daily median. Taking these median values as representative values of the number of close interactions that occur daily and considering that the number of users who participated in the study is 43804 (see Table 6), the average number of daily close interactions can be estimated, which in the case of the EP seat allocation strategy is 0.48 and in the case of MRP is 0.24. Therefore, a 50% reduction is achieved with the MRP strategy. Although the number of events in a day is predominantly one for all types, it should be noted that 40% of the multiple co-presence and close interaction EP events occur between users aged between 15 and 19 years, with what this may imply for scenarios in which travel restrictions are adopted for people in this age group. The impact of a vehicle seating strategy designed to minimize the risk of infection, such as MRP, which was used in the simulations carried out in this study, should be noted. The results show that the number of multiple events decreases significantly when this seating strategy is applied, a clear indicator of how the probability of infection is reduced when efforts are made to maintain a safe distance between passengers in a vehicle. Another relevant conclusion can be drawn from the results shown in Table 9: most of the close interactions – 91% if the EP strategy is applied and 98% if the MRP strategy is applied – consist of a single interaction event. Therefore, measures such as increasing the frequency of bus services or using larger vehicles with higher passenger capacity on routes with higher demand would be effective in reducing the risk of infection. The duration of these cases is summarized in Table 10. Of note is the increase in the median duration for close interactions, which is affected by the minimum threshold of 15 min. It should not be forgotten that the co-presence times are a faithful reflection of users travelling in vehicles at the same time, with the interest that this information may have as a reference point for the planning of operations in a transport system.

The event matrices presented in Sect. 4.3 obtain information about who is in contact with whom in the transport system, which age groups are most frequently involved in contacts, and to what extent they are in contact with other age groups. The co-presence event matrices in Fig. 6(a) and (b) show that the most prevalent users are those between 15 and 19 years of age. This result stems from the fact that this group of users is the most numerous, the group that made the greatest number of trips and, moreover, usually has a significant presence in all time bands, as mentioned above. However, when analyzing the close interaction matrices, when applying either the EP or MRP strategy, the distribution of interactions by age group does not reflect this behavior, since the users that are most involved in close interactions are those between 30 and 59 years of age. This result is consistent with the results presented in Sect. 4.1, specifically with those shown in Table 8, which discusses general aspects of trips made by transport users. It also indicates that there is a greater likelihood of close interactions between users in this age range, which would suggest that these users used public transport more frequently, that is, more trips during the period analyzed, and with similar patterns of use. As shown in Fig. 5(c), users in this age group generally make trips lasting more than 15 min. Therefore, it may be concluded that this is the most exposed group. By comparing the close interaction matrices resulting from application of the EP and MRP seat allocation strategies, it is possible to estimate the effect of a seat allocation strategy designed to minimize close interactions and, therefore, to reduce the risk of contagion. As can be seen, the impact is considerable, as the average number of close interactions varies from a maximum value of 0.200 when applying the EP strategy to a maximum value of 0.08 when applying the MRP policy.

If we compare the behavior of the close interactions based on the contact matrix simulated in this study with the behavior of the close contacts obtained in the studies based on inferred contact networks – as cited in Sect. 2.1 (with the POLYMOD project as a reference) – an important difference becomes apparent. This difference is the absence of a pronounced diagonal with high values in the contact matrices, a diagonal that does appear in the studies based on inferred networks, accompanied by two parallel diagonals. These diagonals reflect, firstly, the tendency of individuals to maintain contacts between their own age group and, secondly, contacts between children and middle-aged adults. However, these tendencies are not observed in the behavior of the close interactions reflected in the results obtained in this study. There are two reasons for this difference. The first is that this study is based on mobility data from anonymous users, which is a representative group of the transport system user population and is not conditioned by people’s willingness to participate in a survey, as is the case in studies based on inferred networks. Because they are automatically recorded events, the matrices reflect a more heterogeneous spectrum of participants, representative of population mobility. The second reason is that the events are not social contacts per se. Co-presence or interaction events are mostly involuntary, and do not have to be directly linked to personal relationships established at home, at school or at work, but rather to the place of residence, routine and habits of the participants. However, the heterogeneous contact behavior observed in this study is in line with the results of Eubank (2004).

It should be noted that the methodology has made it possible, for the use case presented, to have data on interactions with an infection risk for a much higher number of anonymous users than in most of the epidemiological studies referenced in Sect. 2 on related work. Only a few studies based on inferred contact networks have been conducted with a larger number of participating users. Finally, in order to determine the statistical relevance of the results for the population of the geographical area in which the transport system operates, Table 11 shows the number of inhabitants of the island of Gran Canaria in the period studied for each of the age groups,^4^ the number of transport users in the use case in the different categories, and the percentage they represent in each case. It is worth noting that the age group with the highest number of users and the highest mobility in this intercity public transport system – passengers aged between 15 and 19 (see Fig. 5(a) and (d)) – corresponds to around 22% of the population in this age range, and that the next most representative group – passengers aged between 20 and 24 – accounts for 15% of the population. Based on these percentages, it may be concluded that the insights gained will be useful in the study of the factors that influence the transmission of respiratory diseases in public transport systems.

**Table 11:** Population of Gran Canaria and users of the transport system by age group

Age groups	Inhabitants	Users	Percentage
0–14	109,616	1009	0.92
15–19	45,409	10,001	22.02
20–24	45,563	7203	15.8
25–29	51,535	3421	6.63
30–39	121,009	4585	3.78
40–49	152,829	5597	3.66
50–59	137,619	5585	4.05
60–69	91,611	3303	3.6
> = 70	96,040	3100	3.22

The complexity of the proposed methodology can be analyzed from the point of view of the data used and from the point of view of the processes involved. The input data corresponding to the entities described in Sect. 3.1 are commonly used in transport operators’ data models, so this methodology can be applied to a large number of road transport systems. However, data on seating locations in each vehicle, discussed in Sect. 3.2, may not be available, as in the use case where, based on the different bodywork types, the representation described in Table 4 was obtained. With regard to the complexity of the processes, three categories may be identified. The first and second can be framed within the data preparation phase of any data mining project, where most of the resources are usually consumed. In the first category, comprising procedures 1 and 2 as illustrated in Fig. 4, for estimating the destination stop and selecting and loading the transport system graph, it should be noted that the ultimate objective is to have a complete, coherent and robust data structure that allows the events to be extracted and estimated in the most immediate and efficient way possible, a task carried out by procedures 4 and 5 in the same diagram, which fall into the second category. Finally, the third category consists of procedures for extracting information, in the form of diagrams and event matrices, such as those presented in the figures in Sect. 4.

From the point of view of the computational complexity of the proposed methodology, the most complex processes are the process of estimating the destination stop, the simulation of vehicle seat occupancy based on an allocation policy and the generation of interaction events. When analysing the complexity of these processes, it must be taken into account that the implementations make use of the features provided by the database services included in the methodology, namely the transport database and the graph database, along with the RStudio development environment.

In the case of the trip destination stop estimation process described in Algorithm 1 of Sect. 3.2.1, a query made to the transport database yields set Qn_2_, which comprises all stops located at a distance of less than threshold U_p_. The complexity of this algorithm is linear of order n, where n is the size of set Qn_2_. Taking into account the transport network of the use case, and taking 1 km as the value of U_p_, the average number of stops that are at a distance less than U_p_ is 21.3 and the median is 20; therefore, the average size of set Qn_2_ is 21 elements. The best case occurs when the size of set Qn_2_ is the minimum value, where 1 is found to be said value. The worst case occurs when the size of this set is the maximum value of 67.

In the case of the vehicle seat occupancy simulation process described in Algorithm 2 of Sect. 3.2.2, to obtain the minimum potential risk, the set of vehicle seats with the lowest potential risk value and the random selection of one of these seats is performed using RStudio functions. The data on passenger trips in a vehicle journey are obtained through queries to the graph database. As expressed in Algorithm 2, the number of operations to be performed by the seat allocation process depends on three factors: the number of passengers travelling on the route service being processed, the number of seats in the vehicle and the number of seats that make up the affected seats list for each seat, this number being dependent on the safety distance used. The number of stops affects the number of queries made to the graph database, with one query being made for each stop on the route, except for the last stop, as no trips begin there. The purpose of these queries is to determine the number of passengers who start their trip at each stop of the vehicle journey being processed. If N_s_ is the number of stops on the route, then (N_s_-1) queries will be made to the graph database. For each passenger, a seat allocation operation is performed, which involves obtaining the set of seats with the lowest potential risk. Assuming a linear search, if N_a_ is the number of seats in the vehicle, then this process will require N_a_ comparison operations. When assigning the seat to the passenger, the affected seats list must be processed to update the potential risk of each seat in this list. Similarly, when the seat becomes vacant, because the passenger occupying the seat has arrived at the destination stop, the potential risk of the seats that are part of its affected seats list is updated. If N_l_ is the size of the affected seats list, assuming that all seats in the vehicle have an affected seats list with the same number of items, then updating the potential risk of the list will require N_l_ update operations, which will be an addition operation in the case of increased risk and a subtraction operation in the case of decreased risk. Therefore, for each passenger who has travelled on the vehicle journey, (N_a_ + 2N_l_) will be required, where N_a_ is the number of comparison operations and N_l_ is the number of update operations.

Finally, it should be noted that the results presented were obtained according to initial specifications concerning the definition of close contact used in the case of COVID-19 and the seating policies used. In the event that these initial specifications were modified – for example, by changing the definition of close contact – the methodology would not be affected, since it is possible to parameterize all the implementing processes.

## Limitations of the study

The first limitation of this study is that it assumes that there is a risk of infection between two people when they are in close contact. Therefore, the methodology used could only be applied in the case of diseases where the main mode of transmission is close contact, as is the case with COVID-19.^5^ A second limitation is that it is applied in intercity road transport systems and assumes that all passengers are seated; this assumption is not a serious limitation, since standing is usually not permitted for safety reasons. Another limitation is that it is assumed that there is a risk of infection in vehicles when two passengers are on the same vehicle at the same time. Therefore, the presence of two passengers at the same stop on the transport network has not been considered. In the case of intercity public road transport, this limitation is of only relative importance because passengers arrive at a stop a few minutes before catching the vehicle in which they will be travelling, and it is not common for them to spend long periods of time at the stops, and because most of the stops on this type of transport system are located outdoors, thus reducing the risk of infection. The final limitation is that since the passenger’s seat in the vehicle is not known, the location of the passenger was simulated based on a seating allocation policy. The importance of this limitation is also relative, since the objective of the study was to learn on which routes and at what times the risk of infection is greatest. In this study, the policy applied was an EP policy, the aim of which is to approximate the passenger’s seating behaviour.

## Conclusions

This article has presented a novel methodology that aims to provide useful information for epidemiological control in the context of public road transport systems. Specifically, and in line with the work published in this field, the data provided by the methodology make it possible to estimate the time that two users of the public transport system may be in close contact with each other, the frequency with which these contacts occur and which groups of users of different ages are involved. The processes used in the methodology can be run with different initial specifications depending on the desired aspect: epidemiological in relation to the disease scenario, temporal to indicate the period of study, or geographical to specify the area of interest of the transport network. The methodology is based on data mining and considers all the stages required to achieve the proposed goals, including formalisation of the problem, incorporating concepts related to transport activity and epidemiology, and estimation of missing or unknown data based on habitual behaviours observed in the users of transport systems. The design of the proposed methodology makes it possible to obtain useful epidemiological data on a large number of people, based on large sample sizes and obtained over extended periods of time. This allows for comparative analyses of epidemiological data on the populations under study. Another contribution of the methodology is that it is designed to assess the impact of different measures that could be implemented by transport operators to reduce the risk of infection among their users, such as the introduction of seating strategies in vehicles.

The proposal was applied to a real case scenario to illustrate a use case. This involved analysing data from trips made on the intercity transport system of the island of Gran Canaria (Canary Islands, Spain) in the month of December 2019. The results revealed how often users travelled in the period analysed and made it possible to estimate the duration of their trips and how often they travelled with other users in the same vehicle and, additionally, how many pairs of users were within a distance of less than two metres, simulating the passengers’ choice of free seats using two different methods, one more conservative than the other. These data were used to obtain estimates of epidemiological data such as the duration and frequency of potential close contacts that may occur on the transport system each day of the period analysed, and how these contacts are distributed according to different age groups, using the co-presence and close contact matrices. These results were used to identify the age groups most at risk of infection in the period analysed: 40–49, 30–39 and 50–59. It was also possible to estimate the impact of two seating strategies on the vehicles operating in the transport system, one based on observed empirical behaviour and the other aiming to avoid close contacts as much as possible.

Proposed future lines of work include combining the methodology with systems that predict the dynamics of disease spread or study the impact of effective measures to reduce the risk of infection in the context of public transport systems; systems that use hybrid methodologies for decision-making could be of particular interest due to their innovative nature.

## Data Availability

The data that support the findings of this study are available from Salcai Utinsa S. A. (GLOBAL) but restrictions apply to the availability of these data, which were used under license for the current study, and so are not publicly available. Data are however available from the authors upon reasonable request and with permission of Salcai Utinsa S. A. (GLOBAL) (https://www.guaguasglobal.com/).

## Footnotes

1 World Health Organization (2021) Coronavirus disease (COVID-19): How is it transmitted? https://www.who.int/news-room/q-a-detail/coronavirus-disease-covid-19-how-is-it-transmitted. Accessed August 2021.

2 International Transport Forum (2022), Passenger transport, ITF Transport Statistics (database), https://doi.org/10.1787/g2g5557f-en. Accessed July 2022.

3 Centers for Disease Control and Prevention. Appendices: Appendix A Glossary of Key Terms. https://www.cdc.gov/coronavirus/2019-ncov/php/contact-tracing/contact-tracing-plan/appendix.html#contact. Accessed 27 May 2021.

4 Data obtained from the Canary Islands Statistics Institute on 17 June 2021 (ISTAC, http://www.gobiernodecanarias.org/istac/estadisticas/demografia/).

5 World Health Organization: How does COVID-19 spread between people? https://www.who.int/emergencies/diseases/novel-coronavirus-2019/question-and-answers-hub/q-a-detail/coronavirus-disease-covid-19-how-is-it-transmitted. Accessed June, 2022.
